# Trajectories in depressive symptoms and midlife brain health

**DOI:** 10.1038/s41398-024-02883-2

**Published:** 2024-03-29

**Authors:** Christina S. Dintica, Mohamad Habes, Pamela J. Schreiner, Lenore J. Launer, Kristine Yaffe

**Affiliations:** 1 Department of Psychiatry and Behavioral Sciences, University of California, San Francisco, California, CA USA; 2 Neuroimage Analytics Laboratory (NAL) and the Biggs Institute Neuroimaging Core (BINC), Glenn Biggs Institute for Alzheimer’s & Neurodegenerative Diseases, University of Texas Health Science Center San Antonio (UTHSCSA), San Antonio, TX USA; 3 Division of Epidemiology & Community Health, University of Minnesota, Minneapolis, MN USA; 4 National Institute on Aging, Baltimore, MD USA

**Keywords:** Predictive markers, Depression

## Abstract

Depressive symptoms may either be a risk factor or prodromal to dementia. Investigating this association in midlife may help clarify the role of depression in cognitive aging. We aimed to identify trajectories in depressive symptoms in early to mid-life and related cognitive and brain outcomes in midlife. This study includes 3944 Black and White participants (ages 26−45 years at baseline) from the Coronary Artery Risk Development in Young Adults (CARDIA) study with 20 years of follow-up. Depressive symptoms were assessed using the Center for Epidemiological Studies Depression scale at five time points over 20 years. Growth mixture modeling (GMM) was used to identify depressive symptom trajectories. Participants completed a neuropsychological battery 20 years after baseline, including the Digit Symbol Substitution Test (DSST), Rey-Auditory Verbal Learning Test (RAVLT), Stroop Test, Montreal Cognitive Assessment (MoCA), and category and letter fluency tests. A sub-sample of participants (*n* = 662) underwent brain magnetic resonance imaging (MRI) to characterize gray matter volumes and white matter hyperintensities (WMHs). We identified four classes of depressive symptom trajectories: a “declining” class (*n* = 286, 7.3%) with initially high symptoms and subsequent decline, a class with consistently high symptoms (“steady high”; *n* = 264, 6.7%), a class with late increases in symptoms (“increasing”; *n* = 277, 7%), and a class with consistently low symptoms (“steady low”; *n* = 3117, 79.0%). The steady high and the increasing classes had poorer performance on all cognitive tests, while the declining class had poorer performance on the DSST, verbal fluency, and MoCA. Compared to the steady low symptom class, the steady high class had lower volumes in the entorhinal cortex (β: −180.80, 95% CI: −336.69 to −24.91) and the amygdala (β: −40.97, 95% CI: −74.09 to −7.85), the increasing class had more WMHs (β: 0.55, 95% CI: 0.22 to 0.89), and the declining class was not significantly different in any brain measures. Trajectories in depressive symptoms in young to mid-adulthood show distinct cognitive and brain phenotypes in midlife. Steady high depressive symptoms may represent a group that is at risk for dementia, whereas increasing symptoms in midlife may be associated with white matter damage.

## Introduction

Depression and dementia are two prevalent and debilitating conditions that pose significant challenges to global healthcare systems and societies. Studies on the neurobiological basis of depression in late life suggest that depressive symptoms likely serve as both a risk factor and a prodromal symptom of dementia. For instance, growing evidence suggests that depression, which is a treatable condition, is a risk factor for dementia [1]. In a life-course model of the contribution of modifiable risk factors to dementia, the elimination of depression is associated with a 4% reduction in dementia incidence on the population level, exceeding the estimated effects of hypertension (2%), diabetes (1.2), or obesity (0.8%) [2]. However, the mechanisms underlying this link and the temporal relationship between these conditions remain elusive. Clarifying the interplay between depression and dementia is crucial for advancing our knowledge of these complex disorders and developing effective prevention and intervention strategies.

Depressive symptoms in the general population, when monitored over time, display different patterns [3]. Although the majority of individuals have minimal to no symptoms for their adult life, some go through periods of higher symptoms, while others consistently exhibit high number of depressive symptoms [3]. These varying patterns of change may have different associations with brain structure and function in later adulthood, but there is limited evidence to support this hypothesis [4]. Similarly, a 28-year analysis of depressive trajectories revealed that late-onset, but not early-onset, depressive symptoms were a predictor of greater dementia risk [5, 6].

These findings have led to the hypothesis that depressive symptoms could be an early prodromal symptom of dementia or that the two share common causes, rather than depression being a risk factor for dementia [4, 5, 7]. However, to understand the relationship between life course depressive symptoms and cognitive outcomes, studying symptom heterogeneity earlier in life is necessary. This has important implications for preventive strategies to lower the risk of dementia, as depression is a treatable condition.

We previously showed that higher cumulative depressive symptoms from early to mid-life were associated with more advanced brain age based on age-expected atrophy, as well as poorer cognitive function in midlife [8]. Building on those findings, the aims of this study were to (a) to characterize trajectories in depressive symptoms from early adulthood to midlife and (b) investigate whether trajectories in depressive symptoms are differentially associated with cognitive performance in different domains and structural brain differences in midlife.

## Methods

### Study population

The Coronary Artery Risk Development in Young Adults (CARDIA) study is a prospective cohort study investigating the development of and risk factors for cardiovascular disease [9]. Briefly, starting in 1985, 5115 Black and White adults between 18 and 30 years of age were recruited from population-based samples of four US cities (Birmingham, AL; Chicago, IL; Minneapolis, MN; and Oakland, CA). Within each center, recruitment was balanced by race, sex, age, and educational level. Participants completed follow-up examinations every 2 to 5 years for 30 years and depressive symptoms were measured repeatedly from Year 10 (our study baseline) between 1995 and 2015. At each examination, participants provided written informed consent, and study protocols were approved by institutional review boards at each study site and the CARDIA Coordinating Center. Further details regarding the design and recruitment of CARDIA have been previously reported [9]. The full sample consists of the 3944 participants who participated at baseline. All the participants who participated at the study baseline had the assessment of depressive symptoms at baseline and for the subsequent years, 49 (1.3%) at year 5, 101 (2.9%) at year 10, 44 (1.3%) at year 15, and 90 (2.7%), had missing depressive symptom assessment.

### Assessment of depressive symptoms

The participants underwent repeated assessments for depressive symptoms in the past week measured with the Center for Epidemiological Studies Depression scale (CES-D) [10] during each follow-up visit every 5 years. CES-D range is 0−60, with a score of 16 points or more is considered dysthymic. Questionnaire data on antidepressant medication use were collected during the four latest visits (years 2000, 2005, 2010, and 2015).

### Neuroimaging protocol

Brain magnetic resonance imaging (MRI) was conducted at three out of the four CARDIA sites at Year 30 (2015−2016). The MRI sub-sample consists of the 662 CARDIA participants who underwent MRI scanning at Year 30. The MRI scans were acquired on 3 T scanners located at each CARDIA study sites: Siemens 3 T Tim Trio/VB15 platform in Minneapolis and in Oakland and Philips 3 T Achieva/2.6.3.6 platform in Birmingham. Standard quality assurance protocols using phantoms previously developed for the Functional Bioinformatics Research Network (FBIRN) and the Alzheimer’s disease Neuroimaging Initiative (ADNI) were used. Briefly, The MRI Reading Center (RC), located at the University of Pennsylvania, worked in collaboration with the MRI field centers to train technologists to standardized protocols, and transfer MRI data to a central archive located at the MRI RC. To evaluate scanner stability and image distortion prior to site acceptance and quarterly thereafter, each MRI field center followed standard quality assurance protocols developed for the FBIRN, and the ADNI. The following established quality assurance acceptance thresholds were used: FBIRN—Siemens scanners SFNR > 220, RDC > 3.1, Philips scanners SFNR > 220, RDC > 2.4; ADNI—SNR > 300, Maximum Distortion >2.0. Performance across the scanners was acceptable for all sequences. The technical error of measurement, an accuracy index that reflects measurement quality of both acquisition and processing of scans, was estimated from scans of 3 persons measured 3 times in the 3 centers; results were 1.2% for TBV, 27.8% for WHMs [11]. Structural images used for this study were acquired with 1 mm isotropic 3D T1 and T2 sequences. Scan acquisition parameters have been previously described [11], and were processed using previously described methods [12–14]. In brief, structural images were processed using an automated multispectral computer algorithm which classified all supratentorial brain tissue into gray matter, white matter, and cerebral spinal fluid and identifies anatomic regions of interest (ROI). After correction of intensity inhomogeneities [15], a multi-atlas skull stripping algorithm was applied for the removal of extra-cerebral tissues [16]. Each T1-weighted scan was then automatically segmented into a set of anatomical gray matter ROIs using a mutli-atlas label fusion method [17]. The images were visually checked for incidental findings, motion artefacts, and other quality issues. We focused on white matter hyperintensities (WMHs), indicative of vascular insults, total brain volume, and brain volumes in the limbic areas such as hippocampus, entorhinal cortex, and amygdala. The average of the left and right values of each ROI were used to create a single region of interest.

### Cognitive function assessment

A battery of standardized tests to measure cognitive function was included at the Year 30 examination. Cognitive training and certification of CARDIA technicians was performed centrally by CARDIA investigators and coordinating center data quality assurance staff. The Digit Symbol Substitution Test (DSST), a subtest of the Wechsler Adult Intelligence Scale (3rd edition), assesses most prominently visual motor speed, sustained attention, and working memory. The range of scores is 0 to 133, with higher scores indicating better performance. The Stroop Test evaluates the ability to view complex visual stimuli and to respond to one stimulus dimension while suppressing the response to another dimension, an “executive” skill largely attributed to frontal lobe function [18]. The interference score provides a measure of how much additional executive processing is needed to respond to an incongruent trial; thus, a higher interference score indicates worse performance on the task. The Rey Auditory Verbal Learning Test (RAVLT) assesses the ability to memorize and to retrieve words (verbal memory). Results from the long delay (10 min) free recall were used in analyses. The range of scores is 0 to 15, with higher scores indicating better performance. Verbal fluency was assessed using the letter and category fluency tests assessing verbal production, semantic memory, phonemic fluency, and language each over 1 minute, with higher score (combining both tests) indicating better performance. The Montreal Cognitive Assessment (MoCA) measures global cognition by including tests for short-term memory, visuospatial abilities, executive function, attention, concentration, working memory, language, and orientation to time and place. Moreover, we created a cognitive composite score by combining the z-scores (mean 0, SD 1) of the DSST, RAVLT, Stroop, and verbal fluency tests. We defined poor cognitive function using a cutoff ≥1 SD below the CARDIA cohort mean for the cognitive composite score, as previously used in CARDIA [19] and other population-based studies [20].

### Covariates

Demographic characteristics, cigarette smoking (current/former vs never), and alcohol consumption (ml/day) were based on self-report. Annual family income was self-reported in ranges, with the lowest being less than $5000 and highest $100,000 or more, dichotomized as income above or below the median income category ($50,000 through $74,999). Diabetes mellitus at baseline was defined as fasting serum glucose ≥126 mg/dL, oral glucose tolerance test ≥200 mg/dL, glycosylated hemoglobin ≥6.5%, or use of diabetes medications. Body mass index (BMI) was calculated as weight in kilograms divided by height in meters squared. Current history of heart conditions was self-reported and included heart attack, angina, rheumatic heart disease, mitral valve prolapse, or other heart conditions. Hypertension was defined as systolic blood pressure >130 and/or diastolic blood pressure >80 or use of antihypertensive medication. Physical activity was measured with the CARDIA Physical Activity History questionnaire which queries the amount of time per week spent in 13 categories of leisure, occupational, and household physical activities over the past 12 months [21]. Physical activity level was summarized as units of total activity incorporating moderate and high intensity activities. Social network was calculated by summing the reported number of close friends and relatives, with a range from 0 to 24 with a higher score representing a larger social network size. Risky childhood environment was self-reported using the Childhood Family Environment questionnaire, based on the Adverse Childhood Experiences questionnaire [22], which included seven items of verbal, physical, or emotional abuse; substance abuse or violence in the home; and presence of disorganization, and/or lack of engagement or affection in the family or household. Each item is scaled from 1 to 4 (“rarely or none of the time” to “most or all of the time”) [23].

### Analysis

We used growth mixture models (GMMs) to model trajectories of depressive symptoms in the full sample and in the MRI subsample separately. The depressive symptoms assessments were every 5 years, covering 20 years in total (Year 10 in 1995 to Year 30 in 2015). The GMM is a longitudinal form of latent class analysis, using mixed models. The GMMs groups participants into latent classes, on the basis of similarities in their trajectory patterns over time. This is done by fitting an increasing number of curves until an optimal balance between model fit and model complexity is reached. Quadratic models with 1 to 5 classes were fit, and the final model was chosen based on the Bayesian information criterion (BIC), LoMendell-Rubin (LMR) likelihood ratio test, and class size >5%. The BIC indicates the fit of a model, the lower the value, the better the fit of the model is. The LMR test is used to compare model fit between two nested models. The parameter estimates were obtained using maximum likelihood estimation, with standard errors that are robust to non-normality. The quadratic slope variance was fixed to zero. For parsimony, the residual variances assumed to be equal across classes and were allowed to vary over time.

We next tested associations between the trajectory class and cognitive performance at year 30 using linear regression adjusted for age, sex, education, and race in the full sample, and in the MRI sub-sample we used linear regression to test the association between trajectory membership and brain structure volumes, adjusted for age, sex, education, race, intracranial volume, and study site. We further adjusted for income, vascular factors (diabetes, smoking, BMI, heart conditions), and psychosocial factors (social network, and childhood environment). Logistic regression was used to test the odds of poor cognitive performance at Year 30 using the depressive symptoms trajectory classes as the predictor variable. The GMMs were fit using Mplus version 8.2. Further processing of results and multinomial linear regression models were performed using Stata v. 15 and RStudio v. 2022.02. 0.

## Results

### Characteristics of the study population

The majority, 2192 (55.6%), were female, approximately half were of Black race 1923 (48.8%) and the mean age at baseline was 35.0 (SD 3.7) years. The mean CES-D at baseline was 10.7 (SD 8.2). During the follow-up period of 20 years, attrition was 24%. Participants who were lost to follow-up were more likely to be male, of Black race, current/former smokers, more likely to have high blood pressure and diabetes and had higher CES-D at baseline (mean difference 1.29, SD = 0.98). Participants in the MRI subsample were more likely to be of White race, less likely to smoke, have high blood pressure, and had lower BMI; however, these participants did not differ significantly in baseline CES-D (Table 1).

**Table 1:** Baseline characteristics in the overall sample and by trajectory class (*n* = 3944).

Variable mean (sd), *n* (%)	Class 1-Declining (*n* = 286, 7.3%)	Class 2- Steady high (*n* = 264, 6.7%)	Class 3- Increasing (*n* = 277, 7%)	Class 4-Steady low (*n* = 3117, 79%)	*p*
Age	35.8 (±3.8)	35.2 (±3.7)	34.7 (±3.6)	35.1 (±3.6)	0.238
Education (years)	13.1 (±2.3)	13.1 (±2.1)	13.4 (±2.1)	14.1 (±2.2)	<0.001
Female sex %	188 (65.7)	173 (65.5)	179 (64.6)	1652 (53.0)	<0.001
Black Race %	194 (67.8)	169 (64.0)	146 (52.7)	1414 (45.4)	<0.001
Current/former smoker %	148 (52.7)	135 (51.3)	137 (50.0)	1232 (39.7)	<0.001
Alcohol consumption (ml/day)	16.2 (±33.7)	13.4 (±26.2)	9.2 (±16.1)	10.4 (±20.7)	0.877
High blood pressure %	44 (15.7)	48 (18.4)	31 (11.4)	331 (10.7)	<0.001
Heart condition %	38 (13.6)	38 (14.6)	39 (14.2)	291 (9.4)	0.002
Diabetes %	26 (9.2)	15 (5.7)	7 (2.6)	125 (4.0)	<0.001
BMI	28.5 (±7.5)	29.1 (±7.5)	27.8 (±7.1)	27.3 (±6.3)	<0.001
Physical exercise (exercise units)	284.8 (±271)	292.2 (±275.4)	290.1 (±247)	342.0 (±276)	<0.001
Social network	6.9 (6.5)	6.4 (5.6)	6.8 (5.0)	8.3 (6.0)	<0.001
Risky childhood environment	6.8 (5.1)	7.8 (4.9)	6.8 (4.0)	5.8 (3.9)	<0.001

### Heterogeneity of trajectories

**Fig. 1:**
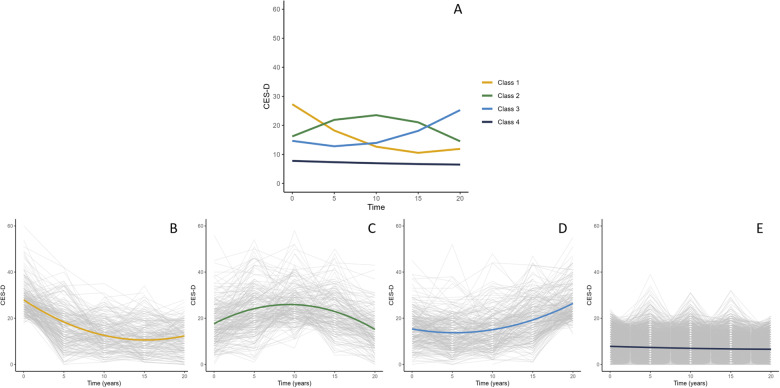
Trajectories of depressive symptoms across 20 years in full sample (*n* = 3944). **A** All trajectories: **B** Class 1-Declining (*n* = 286, 7.3%); **C** Class 2- Steady high (*n* = 264, 6.7%); **D** Class 3- Increasing (*n* = 277, 7%); **E** Class 4-Steady low (*n* = 3117, 79%). CES-D=Center for Epidemiologic Studies Depression Scale.

**Fig. 2:**
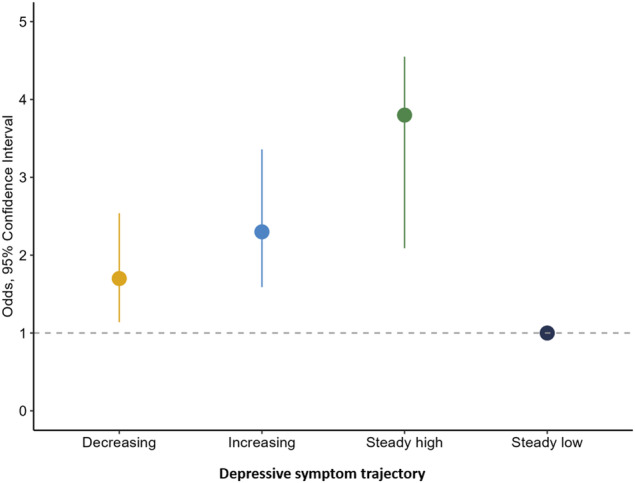
Odds ratio and 95% confidence interval of poor cognitive performance* (1 SD below mean) by depressive symptom trajectory class (*n* = 3944). *Poor cognitive function was defined using a cutoff ≥1 SD below the CARDIA cohort mean for the cognitive composite score.

Quadratic curves were fit for depressive symptom trajectories across 5 assessments spanning 20 years. When fitting models with increasing numbers of classes, the 4-class model provided the best balance between model fit and model complexity, as confirmed by the LMR test (4- vs 5-class model: −2LL(4) = −53158.77, *p* = 0.08). An overview of the model fit criteria is shown in Supplementary Table 1. The best-fitting 4-class model included a class-specific intercept and slope variance. The parameter estimates of the 4-class model are shown in Supplementary Table 2, and the trajectories are depicted in Figs. 1, 2. Class 1 was characterized by an early peak and decline in symptoms (“declining”; *n* = 286, 7.3%); class 2 consistently had high scores (“steady high”; *n* = 264, 6.7%); class 3 had late increases in symptoms (“increasing”; *n* = 277, 7%), and class 4 had consistently low scores (“steady low”; *n* = 3117, 79.0%).

The trajectories in the MRI subsample were replicated, with the 4-class model providing the best balance between model fit and model complexity, as confirmed by the LMR test (4- vs 5-class model: −2LL(4) = −9741.71, *p* = 0.327). The best-fitting 4-class model included a class-specific intercept and slope variance. The parameter estimates of the 4-class model are shown in Supplementary Table 3, and the trajectories are depicted in Supplementary Fig. 1. The declining class was characterized by an early peak and decline in symptoms (“declining”; *n* = 63, 9.5%), class 2 consistently had high scores (“steady high”; *n* = 41, 6.2%), class 3 had late increases in symptoms (“increasing”; *n* = 49, 7.4%), and class 4 had consistently low scores (“steady low”; *n* = 509, 76.9%).

### Factors associated with class membership

Female sex, Black race, lower income, diabetes, heart condition, higher social, strain, and smaller social network increased the likelihood of belonging to the class with declining symptoms relative to the class with steady low symptoms. Female sex, Black race, lower education, lower income, heart condition, smaller social networks, and a riskier childhood environment were more likely to belong to the class with steady high or increasing symptoms relative to the class with steady low symptoms (Table 2).

Within the MRI sub-sample, the factors associated for class membership were similar but associations were attenuated.

### Association between trajectories in depressive symptoms and cognitive performance

Table 2 shows the adjusted mean cognitive test performance according to depressive symptom trajectories. Participants with declining symptoms had significantly lower performance on the DSST, verbal fluency, and the MoCa tests, while the steady high and increasing symptoms classes had lower performance on all cognitive tests compared to the class with steady low symptoms. Moreover, compared to the steady low symptom class, all three classes with higher depressive symptoms had greater likelihood of poor cognitive function (≥1 SD on cognitive composite) at Year 30 (decreasing class OR: 1.70, 95% CI: 1.14 to 2.54; steady high class OR: 3.08, 95% CI: 2.08 to 4.56; increasing class OR: 2.31, 95% CI: 1.59 to 3.36), Fig. 2. Further adjustment for income, smoking, heart condition, diabetes, BMI, high blood pressure, social network, and risky childhood environment produced attenuated but similar results except for verbal fluency, which remained significantly lower only in the class steady high symptoms.

**Table 2:** Adjusted means of cognitive measures by 20-year trajectories in depressive symptom classes in full sample (*n* = 3944).

Cognitive measure	Class 1-Declining (*n* = 286, 7.3%)	Class 2- Steady high (*n* = 264, 6.7%)	Class 3- Increasing (*n* = 277, 7%)	Class 4-Steady low (*n* = 3117, 79%)
DSST	65.46 (63.35 to 67.56)^**^	61.48 (59.36 to 63.61)^***^	64.80 (62.97 to 66.64)^***^	68.77 (68.18 to 69.37)
RAVLT	8.48 (8.05 to 8.92)	7.77 (7.33 to 8.23)^***^	7.92 (7.53 to 8.30)^***^	8.64 (8.51 to 8.76)
Stroop test^a^	22.79 (24.40 to 21.19)	26.51 (28.13 to 24.19)^***^	24.39 (25.78 to 22.99)^**^	22.33 (22.78 to 21.87)
Verbal Fluency	29.90 (28.78 to 31.02)^*^	29.29 (28.18 to 30.42)^**^	29.68 (28.70 to 30.66)^**^	31.19 (30.87 to 31.50)
MoCa	23.31 (22.83 to 23.79)^**^	23.06 (22.57 to 23.54)^***^	23.23 (22.81 to 23.64)^***^	24.05 (23.92 to 24.19)

Adjusted for age, sex, education, and race.

*DSST* Digit Symbol Substitution Test, *RAVLT* Rey Auditory Verbal Learning Test, *MoCa* Montreal cognitive assessment.

^a^Higher score indicates worse performance.

*Significantly different from reference group (Class 4), *p* < 0.05.

**Significantly different from reference group (Class 4), *p* < 0.01.

***Significantly different from reference group (Class 4), *p* < 0.001.

The association between trajectories in depressive symptoms and cognitive performance were similar within the MRI subsample (Supplementary Table 4).

### Association between trajectories in depressive symptoms and brain structure volumes

Table 3 shows differences in brain measures among the depressive symptom classes with the steady low symptom class as reference group. The steady high symptom class had lower volumes in the entorhinal cortex and the amygdala, whereas the class with increasing symptoms had lower total brain volume and higher WMHs. The declining class was not significantly different from the steady low class on any of the brain MRI measures. Additionally adjusting for income, smoking, heart condition, diabetes, BMI, high blood pressure, social network, and risky childhood environment produced attenuated but similar results, however, the steady high class was no longer significantly associated with lower entorhinal cortex.

**Table 3:** Association between 20-year trajectories in depressive symptoms and brain measures in MRI sub-sample (*n* = 662).

Brain Volume (mm^3^)	Class 1-Declining *n* = 49 (7.4%)	Class 2- Steady high *n* = 41 (6.2%)	Class 3- Increasing *n* = 63 (9.5%)	Class 4-Steady low 509 (76.9%)
Total GM volume	1161.36 (−10578.53 to 8255.82)	−7782.28 (−17403.77 to 1838.22)	−9269.25 (−17104.67 to −1433.82)*	Reference
Entorhinal cortex	−115.96 (−261.66 to 29.76)	−180.81 (−336.69 to −24.91)*	−60.57 (−188.06 to 66.91)	Reference
Hippocampus	−59.32 (−242.65 to 124.01)	−183.02 (−379.15 to 13.11)	15.79 (−144.61 to 176.18)	Reference
Amygdala	6.93 (−27.96 to 41.81)	−40.97 (−74.09 to −7.85)*	−10.88 (−39.22 to 17.46)	Reference
Total WMHs	0.90 (−0.02 to 1.82)	0.41 (−0.13 to 0.94)	0.54 (0.21 to 0.87)*	Reference

Adjusted for age, sex, education, race, and, intracranial volume, and scanning center.

*WHMs* White matter hyperintensities, *GM* gray matter.

*Significantly different from reference group (Class 4), *p* < 0.05.

## Discussion

In this large group of community-dwelling middle-aged adults, we identified four trajectories of depressive symptoms over a period of 20 years: those with decreasing, steady high, increasing and steady low depressive symptoms. The decreasing, steady high, and increasing classes all had lower cognitive performance, and higher odds of poor cognitive performance on the composite score at midlife compared with the low symptom group. In the MRI subsample, the steady high trajectory was associated with lower volumes in limbic areas (entorhinal cortex and the amygdala), while the increasing trajectory was associated with higher WMHs, indicative of vascular insults. These findings suggest that low symptoms or a decline in symptoms in midlife may be protective of brain health.

Some studies suggest that older adults may develop cognitive impairment as a result of experiencing depressive symptoms and the pathological correlates of depressive episodes or stress [24, 25], while others argue that depressive symptoms are a prodromal feature of cognitive decline, reflecting a shared underlying pathology [5, 7]. Our findings of poor cognitive performance in all groups showing elevated depressive symptoms, suggests that the higher depressive symptoms may have long lasting effects on cognition even if the depressive symptoms have resolved. This is suggestive of that depressive symptoms are a risk factor and not just a prodrome for cognitive impairment or dementia.

Previous studies in older adults have shown mixed findings regarding the “steady high” depressive symptom phenotype. Some have reported that constant high symptoms were not associated with cognitive performance or higher risk of dementia [4], while other studies have found that this group performed worse on several cognitive tasks [26–28]. Using a cohort of more than 4000 middle-aged and older participants of the Korean Longitudinal Study of Aging, Choi and colleagues identified trajectories of depressive symptoms as low, increasing, moderate declining and high, finding the greatest magnitude of cognitive decline in the high depressive symptoms trajectory and the lowest decline in the low depressive symptoms trajectory [26]. In a recent pooled analysis including 17,556 older adults from the Health and Retirement Study and the English Longitudinal Study of Aging, subthreshold depressive symptoms were associated with a greater rate of cognitive decline, while individuals who show improving depressive symptoms do not exhibit accelerated cognitive decline [27]. Kaup and colleagues found that individuals exhibiting high and increasing depressive symptoms trajectories are at higher risk for dementia than those with consistently minimal symptoms or moderate and increasing symptoms [28], which is in agreement with the findings of this study.

Prior studies in older adults have supported the hypothesis that late-onset depressive symptoms may be an early manifestation of cognitive decline and may reflect vascular disease as the link between depression and dementia [29, 30]. A recent study identified five trajectory groups of depressive symptoms from midlife to old age: consistently low scorers, a subgroup with an early peak in depression scores, intermediate scorers, a late symptom subgroup with an increase in symptoms towards the end of the follow-up period, and consistently high scorers. The late, but not the consistently high scorers, showed higher mean diffusivity, larger volumes of WMHs and impaired executive function. In addition, the late subgroup had higher Framingham Stroke Risk scores throughout the follow-up period, indicating a higher load of vascular risk factors [31]. The findings from this study support previous findings in older adults whereby late onset trajectories have been associated with greater vascular risk factors, and impaired white matter structural integrity. Importantly, we show that increasing depressive symptoms are associated with reduced white matter integrity as early as in midlife, hence the vascular component in increasing depressive symptoms is not limited to late life. Investigating when this relationship emerges is an important next step to understand when the optimal time is to intervene to reduce the depressive burden in individuals with higher vascular risk factors.

Most of the previous evidence for longitudinal variations in depressive symptoms and their relation to cognitive and brain measures has been in older populations. However, studies in older adults are more prone to the reverse causation problem, especially as mild cognitive impairment is often accompanied by an increase in depressive symptoms [32]. Very few studies have characterized depressive symptoms from early to mid-adulthood and their association to midlife cognitive and brain outcomes. Midlife represents a critical period when cognitive trajectories may start to diverge in relation to risk factors; thus, understanding the role of depressive symptoms during this life stage if essential. One study including 1676 expectant parents in their 30 s, found that longitudinal depressive symptoms trajectories over 10 years were only minimally associated with brain health markers (brain volumes and cortical thickness) in middle age [33]. This is contrary to what we found in our study, whereby the steady high depressive symptom group exhibited lower volumes of the amygdala and entorhinal cortex. Previous studies in middle-aged and older adults have shown cross-sectional associations between higher depressive symptoms and neurodegeneration biomarkers in the frontolimbic network including brain areas particularly sensitive to Alzheimer’s disease (AD) [34, 35]. The relationship between depressive symptoms and AD may be partly mediated by neurodegeneration in common brain regions. However, smaller volumes in limbic regions have also been associated with increase in depressive symptoms, therefore the directionality of this association is not clear [36]. There has been recent evidence suggesting that depression has a causal role in AD through Mendelian randomization [37]. Moreover, the authors found that a higher depression polygenic risk score was associated with a faster decline of episodic memory over time [37]. Therefore, individuals with chronic high depressive symptoms may represent an at risk group for AD. This is in line with what we and others have found, whereby higher cumulative depressive symptoms in young to mid adulthood are associated with worse cognitive outcomes in midlife and higher risk of dementia [8, 38, 39].

The strengths of this study include the diverse cohort of young Black and White individuals at enrollment, long duration of follow-up and assessment of depressive symptoms over 20 years, and MRI subsample to detect structural brain differences related symptom trajectories. Limitations of this study include the one-time measure of cognition and brain measures, limiting our ability to evaluate the temporality of depressive symptoms and brain aging, and relatively small class sizes. A limitation of CES-D is the one-week assessment of dysthymia, not sustained depression. Moreover, the CARDIA MRI subsample, compared to the main CARDIA cohort, was more likely to be White, have more years of education, have lower depressive symptoms, and were generally in better health. Therefore, the selectivity of the study sample may limit the generalizability of the findings.

In conclusion, in this large biracial midlife cohort, we found that both chronically high symptoms and increasing depressive symptoms in midlife are related to lower cognitive function and distinct brain signatures, suggesting that midlife depressive symptoms are an important marker for brain health.

### Supplementary information

Supplementary information

## Data Availability

CARDIA data are available on reasonable request from the CARDIA Coordinating Center. CARDIA investigators are eager to collaborate with investigators interested in using CARDIA data. Please see the CARDIA website (cardia.dopm. uab.edu) for publications policies and for a list of CARDIA investigators. CARDIA data are also publicly available on the NIH-supported BioLINCC and dbGaP platforms.
