# Supplementary material for: Trajectories in depressive symptoms and midlife brain health
Source: Transl Psychiatry. 2024 Mar 29;14:169. doi: 10.1038/s41398-024-02883-2 (PMC10980805; doi:10.1038/s41398-024-02883-2)
Supplement: Supplementary file 1 — Supplementary information [file 41398_2024_2883_MOESM1_ESM.docx]

**Supplementary Table 1.** Overview of class enumeration for full sample (n= 3944) and MRI sub-sample (n= 662).

| **Full sample** | | |  | | |
| --- | --- | --- | --- | --- | --- |
| No. classes | No. of  parameters | BIC | Entropy | LMRT  P value | Smallest class size % |
| 1 | 9 | 108308.37 | - | - | - |
| 2 | 13 | 107618.10 | 0.888 | 0.015 | 5.7 |
| 3 | 17 | 106939.99 | 0.875 | 0.001 | 6.5 |
| 4 | 21 | 106491.42 | 0.868 | 0.001 | 5.4 |
| 5 | 25 | 106341.55 | 0.863 | 0.080 | 1.4 |
| **MRI sub-sample** | | |  | | |
| 1 | 9 | 20002.68 | - | - | - |
| 2 | 13 | 19867.36 | 0.921 | 0.004 | 7.3 |
| 3 | 17 | 19716.00 | 0.914 | 0.007 | 7.6 |
| 4 | 21 | 19619.82 | 0.920 | 0.006 | 5.2 |
| 5 | 25 | 19609.30 | 0.911 | 0.327 | 1.2 |

BIC= Bayesian information criterion; LMRT: LoMendell-Rubin likelihood ratio test.

**Supplementary Table 2.** Parameter estimates for depressive symptoms trajectories by latent class membership.*

| Prevalence ([*n*]^a^, %) | **Class 1-Declining**  **(n= 286, 7.3%)** | **Class 2- Steady high**  **(n=264, 6.7%)** | **Class 3- Increasing**  **(n=277, 7%)** | **Class 4-Steady low**  **(n= 3117, 79%)** |
| --- | --- | --- | --- | --- |
|  | Mean (SE) | Mean (SE) | Mean (SE) | Mean (SE) |
| **Fixed effects** |  |  |  |  |
| Intercept | 39.79 | 6.44 | 19.54 | 8.30 |
| Linear annual rate of decline | -14.24 | 11.81 | -6.38 | -0.57 |
| Quadratic annual rate of decline | 1.73 | -2.04 | 1.51 | -0.04 |
| **Random effects** |  |  |  |  |
| Intercept variance | 26.36 | 47.45 | 32.22 | 0.322 |
| Linear slope variance | 0.27 | 0.27 | 0.27 | 0.27 |
| Residual variance Year 0 | 19.88 | 19.88 | 19.88 | 19.88 |
| Residual variance Year 5 | 26.50 | 26.50 | 26.50 | 26.50 |
| Residual variance Year 10 | 23.33 | 23.33 | 23.33 | 23.33 |
| Residual variance Year 15 | 22.34 | 22.34 | 22.34 | 22.34 |
| Residual variance Year 20 | 13.62 | 13.62 | 13.62 | 13.62 |

*n was based on the final class counts of the estimated model. Note that individuals are in fact assigned a probability of class membership.

**Supplementary Table 3.** Parameter estimates for depressive symptoms trajectories by latent class membership in the MRI sub-sample (n=662).*

|  | **Class 1-Declining**  **n= 49 (7.4%)** | **Class 2- Steady high**  **n=41 (6.2%)** | **Class 3- Increasing**  **n= 63 (9.5%)** | **Class 4-Steady low**  **509 (76.9%)** |
| --- | --- | --- | --- | --- |
|  | Mean (SE) | Mean (SE) | Mean (SE) | Mean (SE) |
| **Fixed effects** |  |  |  |  |
| Intercept | 38.99 | 6.28 | 20.38 | 7.77 |
| Linear annual rate of decline | -15.09 | 10.95 | -7.35 | -0.83 |
| Quadratic annual rate of decline | 1.85 | -1.91 | 1.55 | 0.08 |
| **Random effects** |  |  |  |  |
| Intercept variance | 11.23 | 19.25 | 32.56 | 7.06 |
| Linear slope variance | 0.36 | 0.99 | 0.63 | -0.20 |
| Residual variance Year 0 | 14.83 | 14.83 | 14.83 | 14.83 |
| Residual variance Year 5 | 21.06 | 21.06 | 21.06 | 21.06 |
| Residual variance Year 10 | 15.57 | 15.57 | 15.57 | 15.57 |
| Residual variance Year 15 | 19.00 | 19.00 | 19.00 | 19.00 |
| Residual variance Year 20 | 11.13 | 11.13 | 11.13 | 11.13 |

*n was based on the final class counts of the estimated model. Note that individuals are in fact assigned a probability of class membership.

**Supplementary Table 4.** Adjusted means of cognitive measures by 20-year trajectories in depressive symptom classes in MRI sub-sample**.**

| Cognitive measure | **Class 1-Declining** | **Class 2- Steady high** | **Class 3- Increasing** | **Class 4-Steady low** |
| --- | --- | --- | --- | --- |
| DSST | 65.79 (61.61 to 69.97) | 63.11 (58.73 to 67.49)^*^ | 66.16 (62.63 to 69.69) | 69.80 (68.56 to 71.03) |
| RAVLT | 8.94 (8.03 to 9.86) | 8.16 (7.21 to 9.10) | 8.50 (7.72 to 9.27) | 9.09 (8.82 to 9.36) |
| Stroop | 23.99 (20.88 to 27.09) | 25.98 (22.77 to 29.19)^*^ | 25.70 (23.08 to 28.32)^*^ | 21.13 (20.21 to 22.05) |
| Verbal Fluency | 32.04 (29.79 to 34.28) | 31.20 (28.90 to 33.50) | 30.71 (28.79 to 32.63) | 31.65 (30.99 to 32.30) |
| MoCa | 23.72 (22.80 to 24.64) | 23.76 (22.41 to 23.99) | 23.20 (22.41 to 23.99)^*^ | 24.40 (24.13 to 24.67) |

Adjusted for age, sex, education, and race.

^a^ Higher score indicates worse performance.

* Significantly different from reference group (Class 4), p<0.01


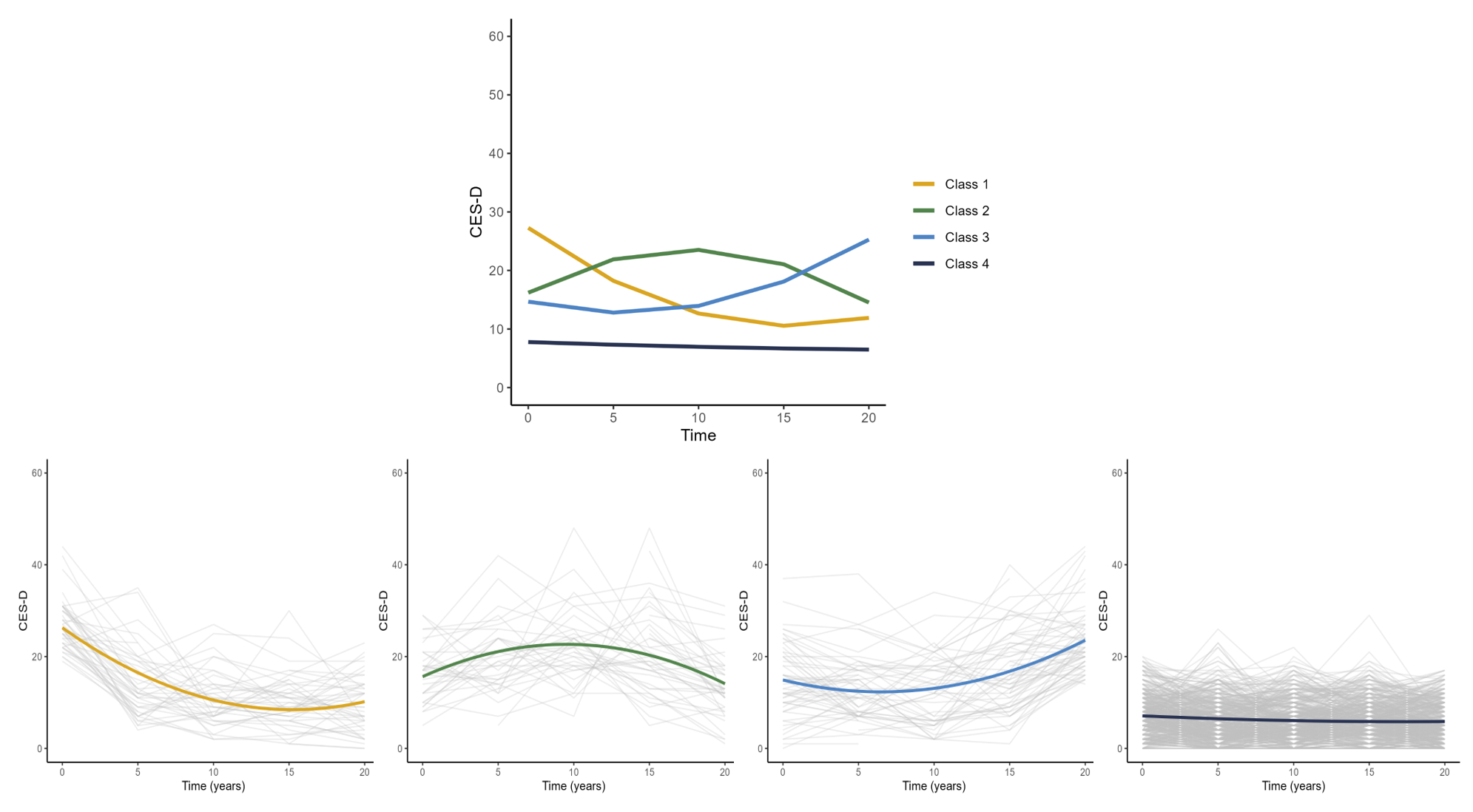
**Supplementary Figure 1.** Trajectories of depressive symptoms across 20 years in the MRI sub-sample (n= 662).

Top left: Class 1-Declining n= 49 (7.4%); top right: Class 2- Steady high n=41 (6.2%); bottom left: Class 3- Increasing n= 63 (9.5%); bottom right: Class 4-Steady low 509 (76.9%). CES-D=Center for Epidemiologic Studies Depression Scale.
